# Microbiota–sphingolipid pathway in generalized epilepsy: evidence from Mendelian randomization and clinical metabolomics

**DOI:** 10.3389/fmicb.2025.1662050

**Published:** 2025-11-28

**Authors:** Jiayin Gong, Mingxing Lin, Lizhen Chen, Wenting Xiong, Yuying Zhang, Changyun Liu, Shenggen Chen, Wanhui Lin, Chaofeng Zhu, Huapin Huang

**Affiliations:** 1 Department of Neurology, Fujian Medical University Union Hospital, Fuzhou, China; 2 Fujian Key Laboratory of Molecular Neurology, Fuzhou, China; 3 Department of Geriatrics, Fujian Medical University Union Hospital, Fuzhou, China

**Keywords:** gut microbiota, epilepsy, mediation Mendelian randomization, metabolomics, sphingomyelin, medium-chain triglyceride diet

## Abstract

**Objective:**

Epilepsy is a complex disorder with growing evidence linking gut microbiota and metabolism, though causal relationships unclear. This study investigated causal effects of gut microbiota on three epilepsy types via metabolic pathways, using Mediation Mendelian randomization (MR), evaluated directional consistency metabolomics of refractory epilepsy (RE) patients before and after medium-chain triglyceride (MCT) diet intervention.

**Methods:**

Two-step MR was applied to summary statistics for 207 species (Dutch Microbiome Project) and 196 species (MiBioGen consortium), evaluating 871 serum metabolites as mediators of three epilepsy types. For validation, directional consistency in metabolomics was conducted on serum samples from 9 RE patients before and after MCT diet intervention.

**Results:**

Only sphingomyelin (SM; d18:0/20:0, d16:0/22:0) and Glycocholate glucuronide (1) were the metabolites significantly associated with three epilepsy types. Mediation MR analysis revealed Mollicutes RF9 had a unidirectional effect via sphingomyelin (d18:1/22:1, d18:2/22:0, d16:1/24:1) modulation (*P* = 0.009). In contrast, Gamma-proteobacteria and Oxalobacter demonstrated bidirectional mediation: via glutamine conjugate of C_6_H_10_O_2_(2) and cerotoylcarnitine (C_26_) (*P* = 0.026 and *P* = 0.033, respectively); while these pathways were protective in mediation, higher abundances were associated with increased risk of generalized epilepsy. Notably, no significant mediators were identified for epilepsy or focal epilepsy. Metabolomics further confirmed MCT diet-induced elevations in 7 specific SM species. Among these, SM (d18:1/36:8) remained statistically significant after Benjamini–Hochberg false discovery rate (BH-FDR) correction. Notably, changes in SM (d18:1/36:8) and SM (d18:1/14:3) were positively correlated with seizure control rates.

**Conclusion:**

This study identifies both unidirectional and bidirectional microbiota–metabolite pathways modulating generalized epilepsy risk, with converging evidence pointing to sphingomyelin as a potential lipid biomarker and therapeutic target.

## 1 Introduction

Epilepsy affects approximately 70 million people worldwide, and remains a major global health challenge (Thijs et al., 2019). Despite advancements in antiepileptic drug development, around one-third of patients remain resistant to existing treatment options, underscoring the critical need to elucidate epilepsy's underlying pathophysiological mechanisms and to identify novel therapeutic targets.

Recent studies suggest that the gut microbiota may play a crucial role in regulating central nervous system (CNS) function via the gut-brain axis (Zhang et al., 2022; Ding et al., 2021). Metabolites derived from gut microbiota, such as bile acids and tryptophan metabolites, can access the CNS through the bloodstream, where they influence microglial and neuronal function and contribute to CNS homeostasis (Deng et al., 2024). Studies have shown that changes in gut microbiota composition are closely linked to systemic metabolic alterations, significantly impacting blood metabolites.

Among non-pharmacological treatments, the ketogenic diet (KD)—a high-fat, low-carbohydrate dietary regimen—has demonstrated substantial efficacy in managing drug-resistant epilepsy. Beyond ketone generation, KD reshapes the composition of gut microbiota by promoting beneficial bacterial populations, with its antiepileptic effects partially attributed to these microbiota-mediated changes (Olson et al., 2018).

The medium-chain triglyceride (MCT)–based KD, in particular, is widely adopted due to its rapid ketone body production and improved gastrointestinal tolerance (Augustin et al., 2018). Although its direct effects on the gut microbiota are less well established, recent animal studies indicate that MCT-KD can alter microbial composition and fecal metabolites (Zhang et al., 2023), supporting its use as a translational model to study microbiota–metabolite interactions in epilepsy.

Although prior two-sample MR studies have identified potential causal links between specific gut microbiota and epilepsy risk (Zeng et al., 2023), the mediating role of microbial metabolites—particularly lipids—remains largely unexplored. The mechanisms by which gut microbiota influence epilepsy through metabolite mediation remain underexplored. To address this, we employed a two-step mediation MR approach to systematically investigate how specific gut microbiota modulate epilepsy risk via metabolite pathways. Furthermore, we performed untargeted serum metabolomics on RE patients before and after MCT-based dietary intervention to evaluate whether observed metabolic shifts support the microbiota–metabolite–epilepsy pathways inferred from mediation MR analysis.

## 2 Materials and methods

### 2.1 Study design

**Figure 1:**
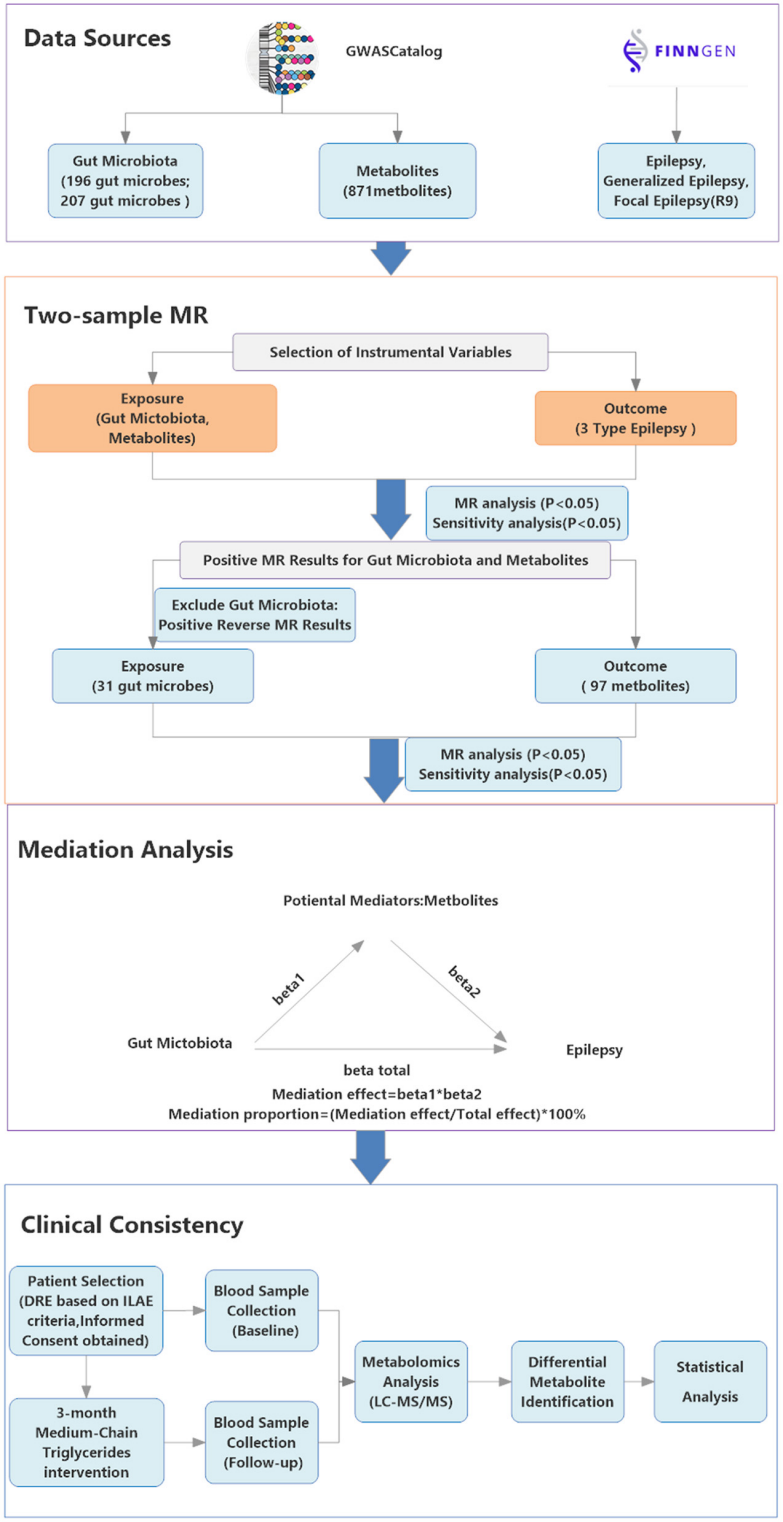
Study Design flowchart. Showing the study's data sources (GWAS Catalog, FinnGen R9), analytical methods (two-step mediation MR, MR with instrumental variables) and clinical consistency process; arrows indicate connections between steps. The GWAS Catalog icon is sourced from its official website (https://www.ebi.ac.uk/gwas/) under the CC BY 4.0 license; the FinnGen icon is sourced from its official website (https://www.finngen.fi/).

See Figure 1 for the detailed study design.

### 2.2 Data sources

#### 2.2.1 Gut microbiota genome-wide association study (GWAS) data sources

Dutch Microbiome Project (DMP) characterized 7,738 Dutch participants by shotgun metagenomics (Lopera-Maya et al., 2022). To minimize cross-platform functional-annotation artifacts, we excluded 205 functional pathways and retained only taxon-abundance traits, resulting in a final set of 207 taxa (5 phyla, 10 classes, 13 orders, 26 families, 48 genera, and 105 species; species-level >50%). Data were downloaded from the GWAS Catalog by GCST90027651 to GCST90027857. MiBioGen included 18,340 participants, whose gut microbiomes were profiled via 16S rRNA amplicon sequencing (Kurilshikov et al., 2021). Starting from 211 core taxa (9 phyla, 16 classes, 20 orders, 35 families, 131 genera), prevalence and annotation filters left 196 taxa for analysis. Data were downloaded from the GWAS Catalog by GCST90016908 to GCST90017118.

Given that 16S rRNA sequencing lacks consistent species-level resolution, we incorporated DMP data (generated via shotgun metagenomics) to provide high-resolution, species-level taxonomic information—consistent with DMP's core advantage of shotgun-based species-level microbiome characterization (Lopera-Maya et al., 2022). To enable side-by-side comparison at shared taxonomic ranks without modifying annotations or removing entries, we compiled Supplementary Table 1-Taxa_alignment. At these comparable ranks, the two datasets collectively cover 5 phyla, 10 classes, 13 orders, 18 families, and 34 genera.

#### 2.2.2 Metabolome-wide GWAS summary data sources

The metabolome-wide GWAS summary statistics are available in the GWAS Catalog under accession numbers GCST90199621 to GCST90201020 (Chen et al., 2023). This dataset comprises 1,091 blood metabolites and 309 metabolite ratios from 8,299 individuals. For this study, we excluded unidentified compounds and metabolite ratios, focusing on the 871 identified metabolites.

#### 2.2.3 Epilepsy GWAS data sources

Epilepsy-related GWAS summary statistics were obtained from the FinnGen consortium's R9 release, encompassing analyses of epilepsy, generalized, and focal epilepsy based on the ICD-10 G40 code (Table 1).

**Table 1:** Data sources for GWAS summary statistics.

**Trait**	**GWAS data sources**	**Samples**	**Case**	**Control**
**Exposure**				
207 microbial taxa	BMP	7,738	/	/
196 microbial taxa	MiBioGen	18,340	/	/
**Mediation**				
Metabolome	GWAS catalog	8,299	/	/
**Outcome**				
Epilepsy	FinnGen (R9)	299,577	11,740	287,837
Generalized epilepsy	FinnGen (R9)	366,832	1,298	365,534
Focal epilepsy	FinnGen (R9)	372,379	6,842	365,537

### 2.3 Mediation Mendelian randomization analysis

#### 2.3.1 Instrumental variables (IVs) selection

The selection of IVs for our study was guided by three principles. First, IVs were selected from GWAS meta-analyses based on a significance threshold of *P* < 1e−5 to capture a comprehensive range of relevant genetic variants (Sanna et al., 2019). Second, we filtered single nucleotide polymorphisms (SNPs) to minimize linkage disequilibrium (LD), applying an R^2^ threshold of < 0.001 within a 10,000-kilobase window to reduce genetic confounding. Finally, each IV was evaluated for strength by calculating the R^2^ and F-statistic, with an F-statistic threshold of >10 to ensure robust instrument strength, thereby avoiding weak instrument bias and enabling valid causal inference (see Supplementary Table 1) (Pierce et al., 2011).

Instruments were constructed and applied independently within each dataset using SNPs specific to the respective source; no cross-dataset pooling of instruments or aggregation of effect sizes was performed.

#### 2.3.2 Statistical analysis

##### 2.3.2.1 MR analysis

In our primary analysis, we employed a two-sample MR approach, with the Inverse Variance Weighted (IVW) method as the core analytical strategy, to investigate the causal effects of gut microbiota and metabolites on epilepsy and its two major subtypes. For MR-derived results, odds ratios (OR) and their corresponding 95% confidence intervals (95% CI) were reported to quantify the strength and uncertainty of the observed associations.

All MR analyses were performed within each dataset using source-specific instruments. For same-named taxa at shared ranks, estimates are reported side-by-side. In this study, all MR analyses used a significance threshold of *P* < 0.05, with Bonferroni corrections and BH-FDR.

##### 2.3.2.2 Sensitivity analysis

To ensure the robustness of our causal inference, we conducted sensitivity analyses, including Cochran's Q-test to detect heterogeneity among estimates (Greco et al., 2015). The heterogeneity Q values, degrees of freedom, and *P-values* for both MR-Egger and IVW methods were calculated to evaluate the stability of the causal estimates. Additionally, the Egger intercept and its significance were examined to detect potential horizontal pleiotropy. These analyses validated the robustness of our results and confirmed that the causal inference was not driven by any single instrument.

##### 2.3.2.3 Bidirectional MR analysis

For the bidirectional MR analysis, we applied reverse MR selectively to those gut microbiota components that demonstrated significant associations with epilepsy and its subtypes in the initial analysis (*P* < 0.05 in the IVW method), with sensitivity analyses confirming these associations (*P* > 0.05).

##### 2.3.2.4 Identification of potential mediators

In this phase, building on the positive findings from previous analyses, we conducted additional MR analyses using gut microbiota as the exposure and metabolites as outcomes. This approach aimed to identify potential mediators for each type of epilepsy.

##### 2.3.2.5 Mediation MR analysis—linking gut microbiota to various types of epilepsy through potential mediators

In further investigations, we evaluated the mediation effects of metabolites on the relationship between gut microbiota and epilepsy, including its subtypes. A two-step MR approach was used: first, the causal effects of gut microbiota on potential mediators associated with three epilepsy traits were estimated (β1). After excluding SNPs correlated with β1, we then assessed the causal effects of these mediators on epilepsy traits (β2), refining the list of mediators. Finally, the total effect (βtotal) of gut microbiota on epilepsy traits was calculated.

We also quantified the direct effects of gut microbiota on epilepsy traits using the formula βdirect = β1 ^*^ β2 and assessed the indirect mediated effects through the coefficient difference method, defined as βtotal - βdirect. The proportion of the mediated effect was calculated by dividing the mediated effect by the total effect, expressed as (βtotal - βdirect)/βtotal.

### 2.4 Pre–post clinical consistency via metabolomics (MCT)

#### 2.4.1 Study population and ketogenic diet intervention

Participants aged 14 to 50 years with drug-resistant epilepsy (DRE) were enrolled based on the 2017 diagnostic criteria established by the International League Against Epilepsy (ILAE). Eligible individuals had failed to achieve seizure control despite treatment with at least two appropriately selected and well-tolerated anti-epileptic drugs (AEDs) at therapeutic doses for a minimum duration of 1 year. Exclusion criteria included severe metabolic disorders, hepatic or renal dysfunction, and any contraindication to ketogenic diet therapy.

The intervention consisted of a medical-grade, medium-chain triglyceride (MCT)–based ketogenic nutritional formulation (*Jing Tong Le*), manufactured under clinical production standards. The formulation contained fat sources such as coconut oil, coconut milk powder, and MCT powder, with a ketogenic ratio of either 3.5:1 or 4.8:1. Participants consumed the product as a partial or total meal replacement over a 12-week period. Regular follow-up visits were conducted to monitor ketosis, seizure frequency, and adverse events.

#### 2.4.2 Sample collection and metabolomics analysis

Fasting venous blood was collected from all 9 patients at baseline (MCT-Pr) and after a 12-week MCT-based ketogenic diet intervention (MCT-Po). Samples were allowed to clot at 4 °C for 30 min and centrifuged at 3,000 rpm for 10 min to obtain serum. The resulting serum was aliquoted into Eppendorf (EP) tubes and stored at −80 °C until analysis. Untargeted serum metabolomics was performed using ultra-high-performance liquid chromatography coupled with tandem mass spectrometry (UHPLC–MS/MS). Quality control included pooled quality control (QC) samples, internal standards, and retention time alignment.

#### 2.4.3 Seizure control rate measurement

To assess seizure control, we used a within-subject design, comparing seizure frequency before and after MCT intervention in the same participants. This allowed us to calculate the Seizure Control Rate as the percentage reduction in seizure frequency following the MCT intervention, using the formula:


$$\begin{array}{l} \text{Seizure Control Rate}=\\ \frac{\text{Pre-intervention frequency – Post-intervention frequency}}{\text{Pre-intervention frequency}}\;\times \text{100\%} \end{array}$$

This approach relies on within-subject comparisons, effectively serving as an internal control, and helps to avoid biases that could arise from inter-individual variability in the absence of an external control group.

#### 2.4.4 Statistical analysis

All pre–post analyses were conducted using R software. Within-subject changes in outcomes were assessed via two-sided paired *t*-tests. The significance was determined by *P-values*, with multiplicity controlled using the BH-FDR method, and results reported as q-values. Standardized paired effect sizes (Cohen's dz) with 95% confidence intervals were derived from bootstrap resampling (2,000 replicates), alongside fold-change and log fold-change.

For SM endpoints, leave-one-out (LOO) sensitivity analyses were performed: the paired *t*-test was refitted iteratively after excluding one participant at a time. The range of *p-values* and Cohen's dz values from the LOO iterations are reported.

Spearman correlation analysis was performed to assess the associations between selected SM species and Seizure Control Rate, using a two-sided rank correlation test. The *p-values* derived from the correlation analysis were not adjusted for multiplicity, and these results should be interpreted as exploratory.

### 2.5 Statistical tools and software

#### 2.5.1 Mendelian randomization analysis

Statistical significance was determined at a threshold of *P* < 0.05 for two-tailed tests. All analyses were conducted in R software (version 4.3.1) using the “TwoSampleMR” package (version 0.5.7) and the “ieugwasr” package (version 0.2.2).

#### 2.5.2 Metabolomics data processing

Data preprocessing and annotation were performed using an in-house R-based program with XCMS and the BiotreeDB (V3.0) database.

#### 2.5.3 Statistical software

GraphPad Prism (Version 10.1.2) was used for the calculation of correlation analyses.

## 3 Results

### 3.1 Details of IVs

We finalized the identification of IVs across different data sources, which included: 1,913 IVs from the BMP for gut microbiota; 2,539 IVs from MiBioGen; 21,833 IVs for metabolites. For detailed information on these IVs, refer to Supplementary Table 1.

### 3.2 Two-sample MR analysis of biological factors on epilepsy, generalized epilepsy, and focal epilepsy, and reverse analysis on gut microbiota

**Figure 2:**
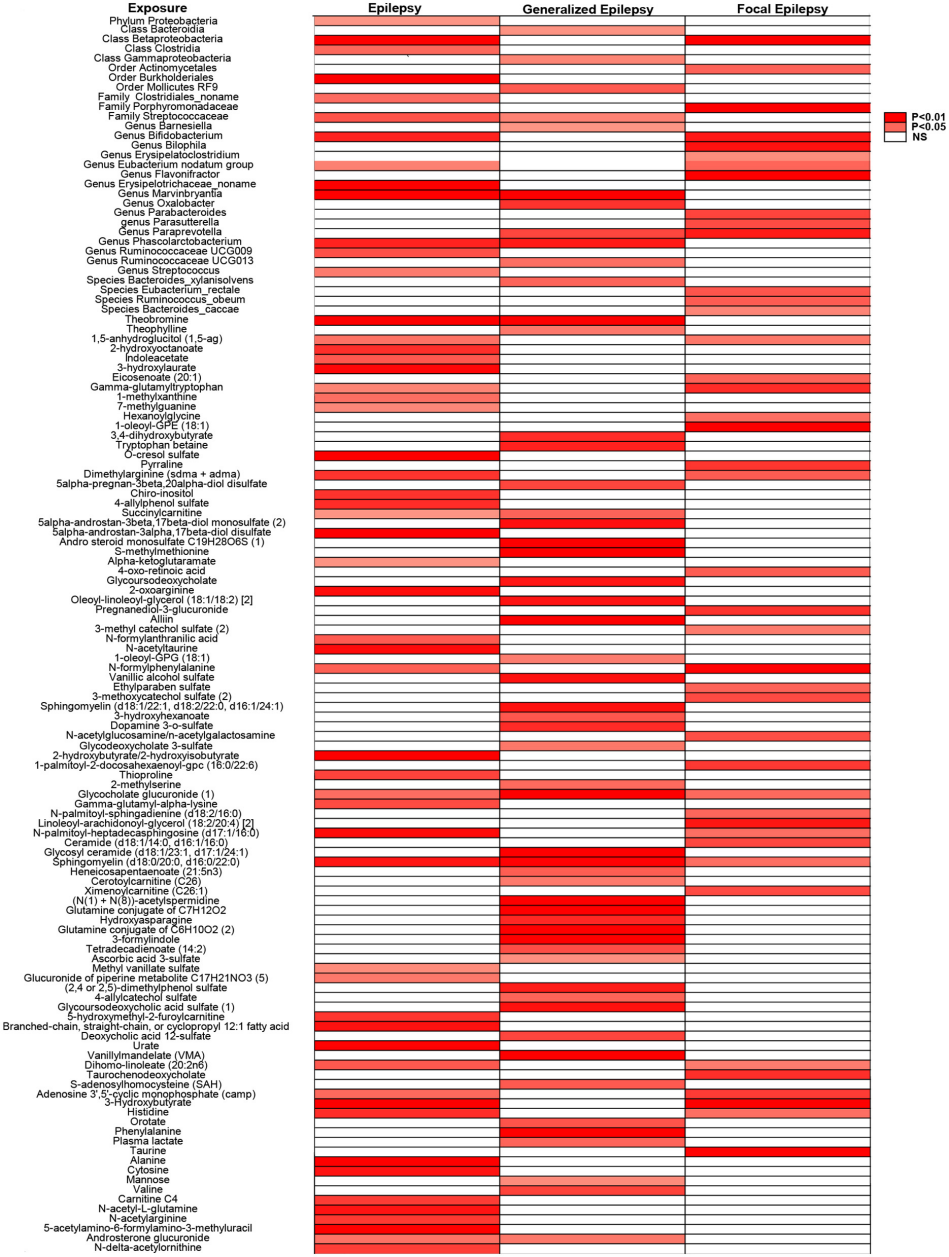
Heatmap of combined causal impacts assessed by the IVW method.

We conducted MR analysis with a significance threshold of *P* < 0.05, complemented by sensitivity analyses (*P* > 0.05 for pleiotropy). This approach identified 31 unique gut microbiota (deduplicated across categories for reporting) and 97 metabolites potentially influencing each type of epilepsy. After Bonferroni corrections (Metabolites: α = 5.74 × 10^−5^; DMP taxa: α = 2.42 × 10^−4^; MiBioGen taxa: α = 2.55 × 10^−4^), no tests remained significant. Accordingly, we report and interpret *P-values* as the primary metric and treat all signals as exploratory. For display only, gut taxa with the same name recurring across categories were label-collapsed to a single marker in Figure 2.

Notably, among the 871 metabolites, only sphingomyelin (d18:0/20:0, d16:0/22:0) and Glycocholate glucuronide (1) were significantly associated with all epilepsy subtypes. Subsequently, reverse MR analysis was performed on gut microbiota with significant forward associations to evaluate potential bidirectional effects. Betaproteobacteria, Phascolarctobacterium, and Ruminococcaceae UCG-009 were associated with epilepsy, while Streptococcaceae and Marvinbryantia showed associations with generalized epilepsy. These analyses are essential for ensuring that future mediation studies include only microbiota without bidirectional effects. The complete dataset is provided in Supplementary Table 2.

### 3.3 Identification of potential mediators using selected positive results

**Figure 3:**
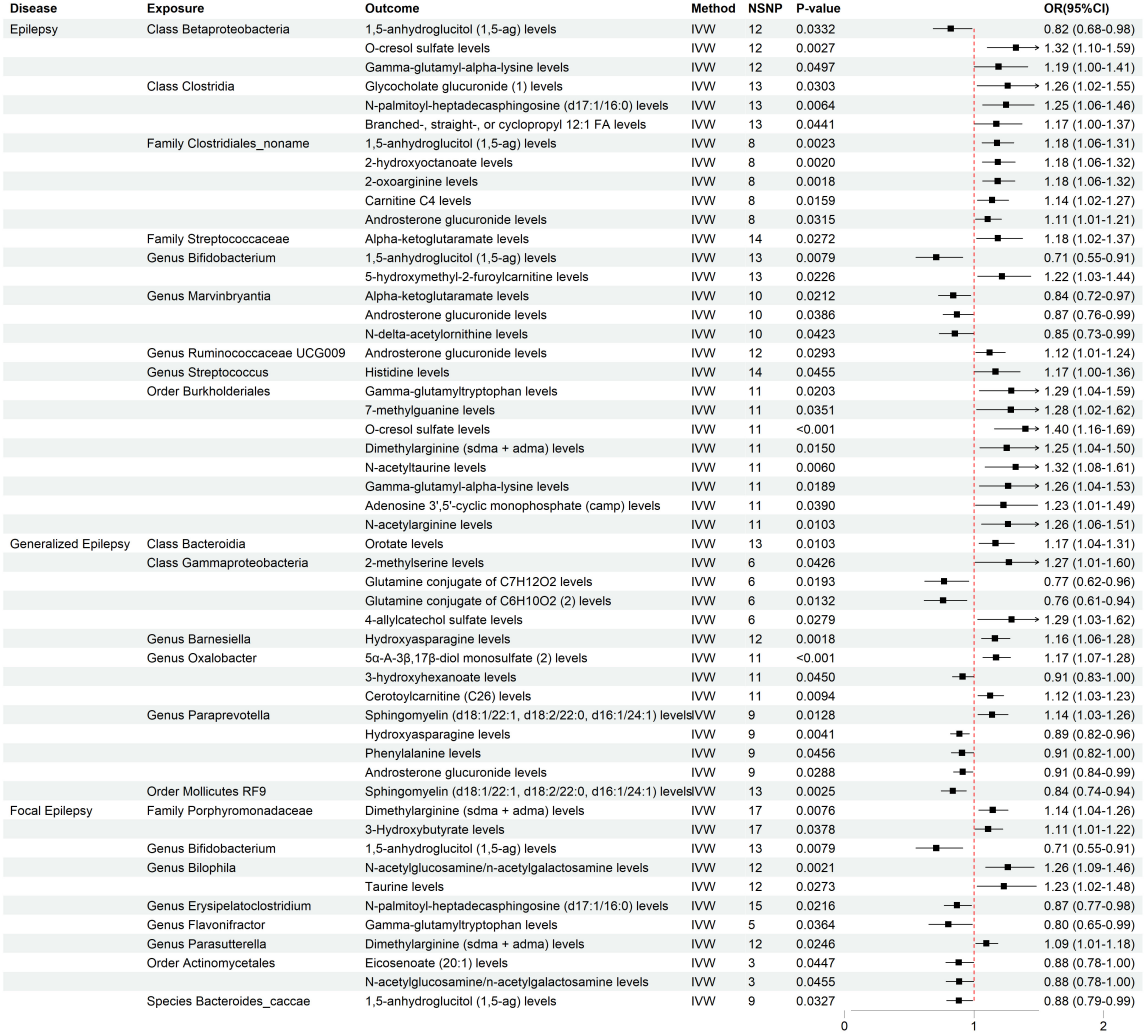
Forest plots of potential mediators identified via MR.

We identified 20 potential mediators in epilepsy, 17 in generalized epilepsy, and 8 in focal epilepsy (Figure 3). These metabolites are involved in various metabolic pathways, including energy metabolism, lipid metabolism, and amino acid metabolism, and may influence the risk of epilepsy through their modulation. Detailed information about the identified mediators is provided in Supplementary Table 3.

### 3.4 Outcomes of mediation analysis in epilepsy traits

Our mediation analysis revealed that, upon reassessment, previously identified mediators such as Hydroxyasparagine, 3-hydroxyhexanoate, and 5alpha-androstan-3beta, 17beta-diolmonosulfate (2) no longer exhibited significant effects in generalized epilepsy. Similarly, Androsterone glucuronide and Branched-chain, straight-chain, or cyclopropyl 12:1 fatty acid also failed to show significant impacts on epilepsy traits (Supplementary Table 4). We then conducted a more comprehensive mediation effect analysis across all mediators. Ultimately, three metabolites were identified as having significant mediation effects specifically associated with generalized epilepsy (Table 2), as follows:

**Table 2:** Positive mediation effects of gut microbiota on generalized epilepsy.

**Gut microbiota**	**Potential mediators**	**Mediated effect**	**Mediated proportion**	***P***
Mollicutes RF9	Sphingomyelin (d18:1/22:1, d18:2/22:0, d16:1/24:1) levels	0.057 (0.015, 0.099)	14.4% (3.68%, 25.2%)	0.009
Gamma-proteobacteria	Glutamine conjugate of C_6_H_10_O_2_(2) levels	−0.151 (−0.285, −0.018)	−21.1% (−39.6%, −2.49%)	0.026
Oxalobacter	Cerotoylcarnitine (C_26_) levels	−0.020 (−0.038, −0.002)	−7.44% (−14.3%, −0.587%)	0.033

**Mollicutes RF9** influenced generalized epilepsy risk through decreased levels of sphingomyelin (d18:1/22:1, d18:2/22:0, d16:1/24:1), with a mediation effect was 0.057 (95% CI: 0.015 to 0.099, *P* = 0.009), accounting for 14.4% (95% CI: 3.68% to 25.2%).

**Gamma-proteobacteria** modulated generalized epilepsy risk via reduced levels of the glutamine conjugate C_6_H_10_O_2_(2), showing a mediation effect of −0.151 (95% CI: −0.285 to −0.018, *P* = 0.026), accounting for −21.1% of the total effect (95% CI: −39.6% to −2.49%).

**Oxalobacter** affected generalized epilepsy risk through increased levels of cerotoylcarnitine (C_26_), with a mediation effect of −0.020 (95% CI: −0.038 to −0.002, *P* = 0.033), accounting for −7.44% of the total effect (95% CI: −14.3% to −0.587%).

**Figure 4:**
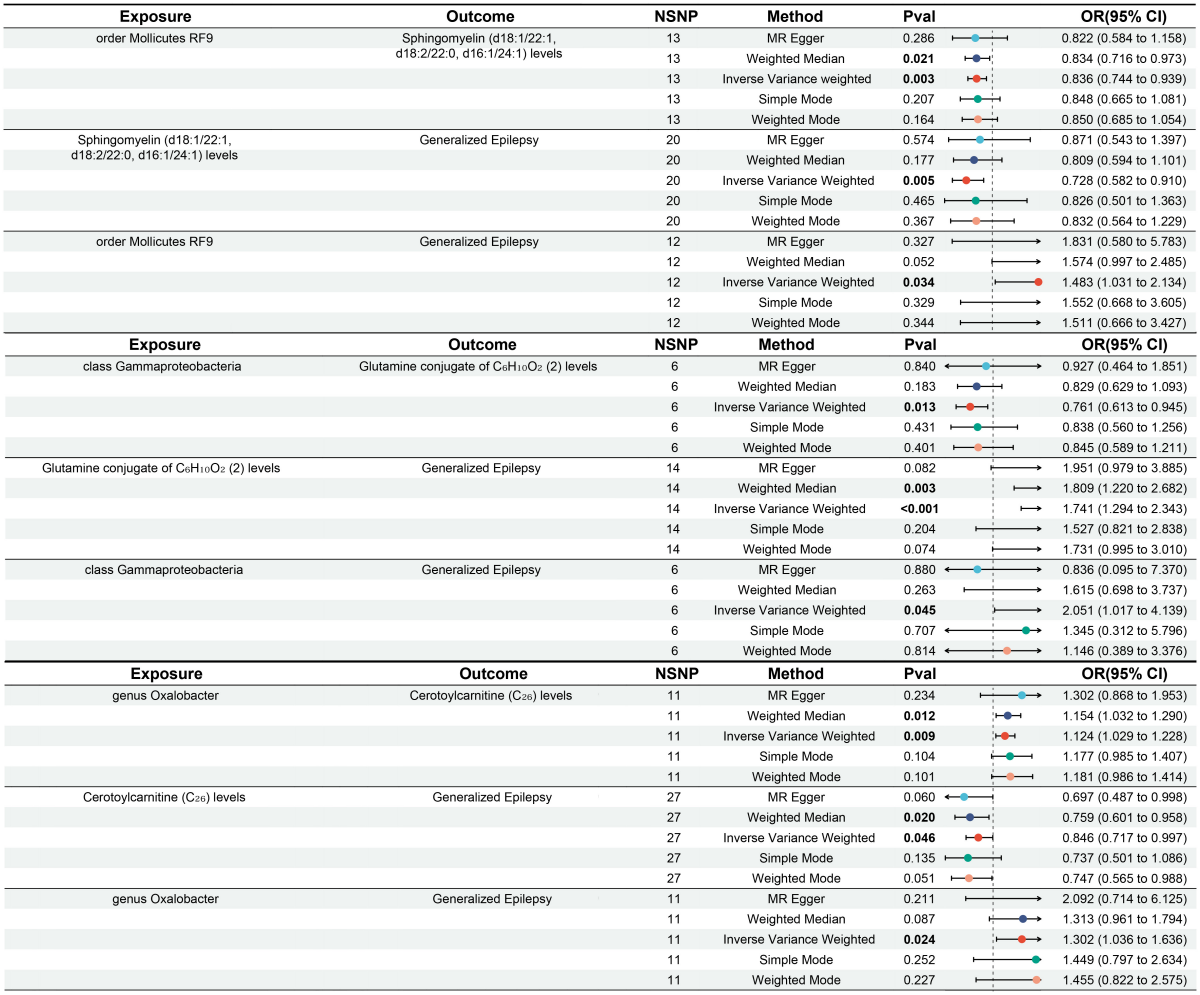
Forest plots of Mendelian MR results for three microbial taxa and associated metabolites related to generalized epilepsy.

No significant mediators were identified for epilepsy or focal epilepsy. The two-step significant MR results are presented in Figure 4. For detailed data, please refer to Supplementary Table 5.

### 3.5 Metabolomics analysis

**Figure 5:**
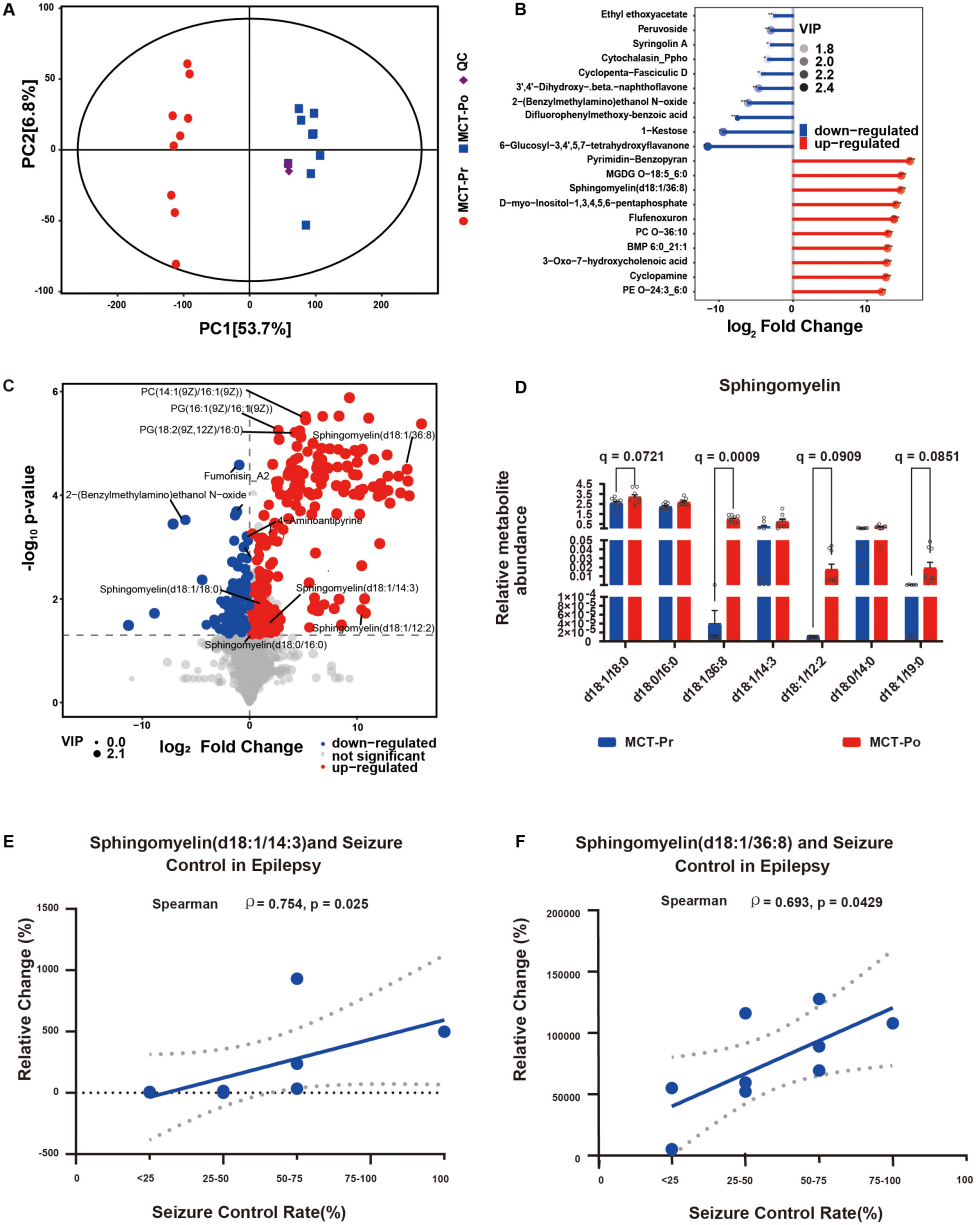
Integrated metabolomic analysis of serum samples from DRE patients before and after MCT-based dietary intervention. **(A)** PCA score plot of serum samples from MCT-Pr and MCT-Po groups. **(B)** Volcano plot showing significantly altered serum metabolites between MCT-Pr and MCT-Po groups. **(C)** Matchstick of differential metabolites between MCT-Pr and MCT-Po groups. Dot size indicates Variable Importance in Projection (VIP) score. Features were selected based on VIP > 1 and *P* < 0.05. **(D)** SM species in MCT-Pr and MCT-Po (*n* = 9). Mean ± SD. Paired *t*-tests; BH-FDR within panel (*q-values*). **(E**, **F)** Spearman correlation between seizure control rate and relative changes in SM species following MCT intervention.

All nine participants completed the 12-week MCT intervention with good compliance and no serious adverse events. Fasting blood β-hydroxybutyrate levels were maintained within the target range of 0.5–1.0 mmol/L. Metabolomic analysis of fasting serum from 9 DRE patients revealed significant metabolic alterations following MCT dietary intervention. Principal component analysis (PCA) revealed a distinct separation between MCT-Pr and MCT-Po groups along PC1 (explaining 53.7% of the total variance), while QC samples clustered tightly, confirming analytical robustness (Figure 5A). Among the significantly altered metabolites, SM (d18:1/36:8) was markedly upregulated after MCT intervention (Figure 5B). Volcano plot displaying significantly altered metabolites following MCT intervention, with a predominance of upregulated sphingomyelin species (Figure 5C). Relative abundance of multiple sphingomyelin species in MCT-Pr and MCT-Po groups. Overall, seven SM species increased after MCT with significant paired *t*-tests (all *P* < 0.05); after BH-FDR within the SM panel, only SM (d18:1/36:8) remained significant (*q* < 0.05) (Figure 5D). Furthermore, Seizure control was assessed based on the Seizure Control Rate, calculated by comparing seizure frequency before and after the MCT intervention. Correlation analysis revealed significant positive associations between seizure control rate and relative increases in specific sphingomyelin species (Figures 5E, F). For pre-specified SM endpoints (and all BH-FDR-significant metabolites), we report effect sizes with 95% CIs and q-values; leave-one-out (LOO) sensitivity indicated no single-subject dominance. The relative abundance of altered sphingomyelins and corresponding seizure frequency changes are summarized in Supplementary Table 6.

This heatmap illustrates the biological factors associated with epilepsy and its subtypes, identified through MR analysis with a significance threshold set at *P* < 0.05. Sensitivity analyses were conducted to ensure pleiotropy, with *P* > 0.05 considered non-significant. Same-named labels are merged for display only; no statistical pooling.

The forest plots display the mediators that showed statistically significant effects, along with their odds ratios (ORs) and confidence intervals (CIs). NSNP, number of instrumental SNPs.

## 4 Discussion

This study utilized mediation MR to uncover the regulatory roles of specific gut microbial taxa in generalized epilepsy risk via serum metabolites. The analysis identified both unidirectional and bidirectional effects, indicating the existence of distinct causal pathways within the microbiota–metabolite–epilepsy axis.

Given that this mediation analysis involves multi-omics data, applying strict multiple testing corrections could significantly reduce statistical power (Ma et al., 2025; Wen et al., 2024; Fan et al., 2025), accordingly, no estimates in our study survived Bonferroni correction and BH-FDR. While these methods help reduce false positives, the core advantage of mediation MR lies in its ability to decompose both direct and indirect effects, with the IVs being rigorously selected to effectively reduce false positive risks (Liu et al., 2025). In exploring complex disease mechanisms and biomarker pathways, excessive correction could remove potentially important signals (Ma et al., 2025; Wang et al., 2023). Our sensitivity analyses confirmed the robustness of the results (Liu et al., 2025), thus retaining the original *P-values* avoids over-correction and ensures that valuable biological signals are preserved.

Firstly, *Mollicutes* RF9 demonstrated a unidirectional regulatory mechanism, increasing generalized epilepsy risk by lowering sphingomyelin levels. In particular, SM (d18:0/20:0, d16:0/22:0) was the metabolite significantly associated with all three epilepsy types in our MR analysis, suggesting a core pathogenic role for this lipid class. SM, essential for neuronal membrane integrity and stability (Kang and Klauda, 2015), has been closely linked to epilepsy risk in both human and rodent models (Ma et al., 2007; Kunduri et al., 2018). The unique characteristics of *Mollicutes*, such as the absence of a cell wall and a small genome (Trachtenberg, 2005), may contribute to its classification and pathogenic processes through its distinct structural features and functions (Worliczek et al., 2007; Hackett et al., 1987), potentially elevating generalized epilepsy risk.

Furthermore, in patients with super-refractory status epilepticus (SRSE) receiving KD treatment, elevated serum SM levels have been observed (Dickens et al., 2023). Mechanistically, KD exerts anti-epileptic and neuroprotective effects by increasing β-hydroxybutyrate and regulating SM metabolism (Dabke et al., 2020; Rugieł et al., 2024). Notably, SM can protect membrane free cholesterol from free radical-mediated oxidation, which is attributed to its unique molecular structure and high affinity for sterols (Sargis, 2006).

Although the mediated proportion for SM is 14.4% (3.68%−25.2%), indicating a statistically significant yet only partial pathway from Mollicutes RF9-SM-generalized epilepsy [mediation effect = 0.057 (0.015–0.099)], approximately 85.6% of the total effect is transmitted outside this metabolite pathway—either via a direct microbiota-epilepsy effect (Liu et al., 2024) or through other mediators (additional metabolites, inflammatory cytokines, and immune regulation) (Li et al., 2023; Zhao et al., 2025; Wang et al., 2024).

However, our mediation MR uses a two-step MR framework to assemble the microbiota-metabolite-epilepsy causal chain. Although IV assumptions hold within links, chain-level inference assumes the metabolite fully transmits the microbiota's effect. Because SM are not exclusively microbially determined (Rohrhofer et al., 2021) and metabolic networks include host-intrinsic synthesis (He et al., 2025), SM can exhibit dysregulation within the CNS independent of gut input (Yan et al., 2025). This implies that host- and microbiota-derived routes may run in parallel or interact (Rohrhofer et al., 2021), thereby introducing residual confounding.

Accordingly, we used Pr/Po-MCT serum metabolomics as a clinical, directionally consistent line of evidence to strengthen translational interpretation.

Clinical metabolomics findings were directionally consistent with the MR-based inference. Seven sphingomyelin species increased after MCT (all *P* < 0.05), with SM (d18:1/36:8) remaining significant after BH-FDR (*q* < 0.05). Notably, increases in SM (d18:1/36:8) and SM (d18:1/14:3) were positively correlated with seizure control rates, reinforcing their relevance as seizure-modifying lipids. These results support the hypothesis that sphingomyelin plays a neuroprotective role and can be modulated by dietary-microbiota interactions (Zhang et al., 2023; García-Belenguer et al., 2023), offering a translational bridge between host genetics and modifiable environmental interventions.

Although the SM subtypes identified via MR and metabolomics differ structurally, these discrepancies likely stem from differences in temporal resolution and detection methodologies, rather than from conflicting biological functions. GWAS can capture genetic loci that regulate metabolite variation (Yang et al., 2024), providing a basis for identifying ceramide backbone subtypes with strong heritability (Cresci et al., 2020). In contrast, LC-MS/MS–based metabolomics techniques exhibit high sensitivity for the accurate detection of acyl-chain-modified variants in biological samples (Zhang et al., 2024). Structural determinism further supports this stratification: the sphingoid backbone is regulated by evolutionarily conserved biosynthetic pathways (Hornemann et al., 2009), whereas phospholipid acyl-chain diversity serves as a plastic interface, adapting to environmental inputs and modulating membrane composition (Chwastek et al., 2020).

Despite their structural diversity, all SM species share a highly conserved sphingosine domain, which may underlie their functional convergence in epilepsy-related mechanisms (Velazquez et al., 2024). This “core domain” determines the high affinity between SM and cholesterol, and this interaction forms the basis of lipid rafts in cell membranes (Barenholz, 2004). This structural conservation likely accounts for the consistent associations observed across MR and metabolomic platforms. Moreover, the upregulation of specific SM subtypes following MCT intervention and their positive correlation with seizure control further supports their biological relevance as potential therapeutic targets. Collectively, sphingolipid metabolism emerges as a central metabolic axis that integrates genetic and environmental signals in epilepsy, warranting further investigation into whether distinct SM subtypes exert their effects through shared or divergent molecular pathways.

In our bidirectional mediation analysis, both Gamma—proteobacteria and Oxalobacter regulate the risk of generalized epilepsy through a “microbiota-metabolite” mediating mode, and both exhibit condition-dependent effects: on one hand, although Gamma—proteobacteria is associated with an increased risk of epilepsy (Wei et al., 2023), it can influence generalized epilepsy by reducing the glutamine conjugate C_6_H_10_O_2_(2). On the other hand, although genetically predicted increased abundance of Oxalobacter is associated with an elevated risk of epilepsy, it can exert a protective effect by increasing the level of cerotoylcarnitine (C_26_).

This paradox—where a risk-associated bacterium modulates a metabolite to exert a protective effect—highlights the complexity of microbiota–metabolite interactions, underscoring the need to consider both microbial function and ecological context when targeting the microbiome for generalized epilepsy management.

The observed microbial-metabolic regulatory patterns may intersect with known effects of KD. Prior studies have shown that KD enriches beneficial bacteria, such as Akkermansia muciniphila and Parabacteroides, and modulates neuroactive metabolites like γ-aminobutyric acid (GABA) (Olson et al., 2018). The observed consistency between our MCT intervention data and previously reported microbial-metabolic pathways (García-Belenguer et al., 2023) highlights the relevance of microbial modulation strategies in epilepsy management. Modifications induced by the modified Mediterranean ketogenic diet (MMKD) may exert regulatory effects on the gut mycobiome, modulate fungal metabolites, and further impact the host's metabolic health (Nagpal et al., 2020), fungi and their metabolites may exert potential therapeutic effects in epilepsy by exerting anti-inflammatory activities, scavenging oxidative stress, and regulating neurotransmitter levels (Sanyasi, 2025; Abd El-Rahman et al., 2023). KD may reshape the gut microbiota, dampen virus-induced neuroinflammation, and recalibrate immunometabolic integration via γδ T-cell activation and ketone-body production, thereby mitigating virome-related effects on epilepsy (Goldberg et al., 2019; Shaheen, 2021; Wouk et al., 2021; Shan et al., 2023).

These studies emphasize that when investigating the impact of the gut microbiota on host metabolism, not only the role of bacteria but also that of fungi and viruses should be taken into account, especially in the context of dietary interventions like the KD.

While significant mediators were identified for generalized epilepsy, no such associations were found for overall or focal epilepsy. This may be due to methodological constraints, such as small sample sizes, measurement variability, and the complex, possibly bidirectional nature of gut microbiota–metabolite interactions. These factors may obscure true effects and highlight the limitations of current models in capturing the full complexity of the gut–brain axis.

## 5 Limitations

Although our findings emphasize the potential role of gut microbiota in generalized epilepsy, several limitations warrant consideration. The possibility of sample overlap in GWAS datasets, lack of multiple testing correction, and cohort homogeneity may affect the robustness and generalizability of the results. Moreover, the lifelong nature of genetic exposures limits the direct clinical translatability of MR-based inferences. The clinical arm lacked paired microbiome profiling and a control group, and the sample size was small (*n* = 9); accordingly, it was not designed to validate a full microbiota-metabolite-seizure causal pathway.

## 6 Future directions

Larger and more diverse cohorts are needed to confirm the microbiota–sphingomyelin pathways and delineate the dynamic contribution of sphingolipids to seizure modulation in epilepsy. Within a multivariable MR framework, concurrent profiling of the microbiome (including the virome and mycobiome) and metabolome—combined with colocalization analyses and non-linear modeling—should be applied to distinguish microbiota-dependent from host-dependent pathways and quantify their interactions. Additionally, longitudinal study designs with paired microbiome–metabolome profiling, alongside mechanistic investigations, will be essential to establish the causality of the microbiota–sphingomyelin axis and validate its clinical relevance for epilepsy management.

## 7 Conclusion

This study identifies distinct unidirectional and bidirectional microbiota–metabolite pathways associated with the risk of generalized epilepsy. Despite methodological differences between MR and metabolomics platforms, both converge on sphingomyelin as a key mechanistic biomarker, highlighting the potential for microbiota–lipid–based personalized interventions.

## Data Availability

The datasets presented in this study can be found in online repositories. The names of the repository/repositories and accession number(s) can be found in the article/Supplementary material.
